# Supplementary material for: Silent voices: Uncovering women’s absence in veterinary surgery publications
Source: PLoS One. 2025 Aug 14;20(8):e0330392. doi: 10.1371/journal.pone.0330392 (PMC12352642; doi:10.1371/journal.pone.0330392)
Supplement: S1 Table — (DOCX) [file pone.0330392.s001.docx]

**Supplementary Data 1: Overview of the selection criteria for the most relevant models**

| Model Parameters | Tjur’s R^2^ | Akaike’s Information Criterion | Mean RMSE across 10 crossvalidations | Bayes R^2^ | Bayesian Estimations Converged? | Bayesian Indices Reliable? | Comments |
| --- | --- | --- | --- | --- | --- | --- | --- |
| Year, Author Place, Surgical Emphasis, Animal Size | 0.05 | 10294 | 1.12 (min: 1.07, max: 1.18) | 0.05 | All | All | Animal size had an alpha > .01, or had probability of insignificance |
| Year, Author Place, Surgical Emphasis, Animal Size, Year x Author Place, Year x Surgical Emphasis, Year x Animal Size, Author Place x Surgical Emphasis, Author Place x Animal Size, Surgical Emphasis x Animal Size | 0.06 | 10302 | 2.80 (min: 1.16, max: 5.43) | NA, Model unreliable | Not all | Not all | Model parameters rank deficient and Bayesian model failed to converge |
| Year, Author Place, Surgical Emphasis, Animal Size, Year x Author Place | 0.05 | 10289 | 1.12 (min: 1.07, max: 1.18) | 0.05 | All | All | Year x Author Place is an interesting interaction to retain, animal size had an alpha > .01 or a probability of insignificance |
| Year, Author Place, Surgical Emphasis, Year x Author Place, Year x Surgical Emphasis, Author Place x Surgical Emphasis | 0.05 | 10287 | 1.12 (min: 1.06, max: 1.18) | 0.05 | All | All | Interactions with Surgical Emphasis have an alpha > .01 or a probability of insignificance |
| Year, Author Place, Surgical Emphasis, Year x Author Place | 0.05 | 10292 | 1.12 (min: 1.07, max: 1.18) | 0.05 | All | All | All fixed effects and interactions are significant or have a probability of significance. Model retained. |

We elected to run each model as a frequentist binary logistic regression, where 95% Confidence Intervals (CIs) and p-values were computed using a Wald z-distribution approximation, and also through a Bayesian estimation of probability using a Markov chain Monte Carlo (MCMC) algorithm to sample from a probability distribution and update probabilities (posteriors) based on observed data (4,5).

For each model, we considered a range of criteria. For the frequentist model we examined each model’s cumulative explanatory power via Tjur’s R^2^ (1), the Akaike’s Information Criterion, and utilised k-fold cross validation via the ‘rsample’ package (2) to create 10 validation and training datasets where each data point has been present in both the validation and training dataset. For each cross-validated model we calculated the average Root Mean Square Error (RMSE)(3). The RMSE represents how big the ‘typical’ prediction error is for any given model, in the same units as the response variable. A smaller RMSE reflects a more accurate model. For the Bayesian models we compared the Bayes R^2^ as a measure of the model’s explanatory power. We also examined the alpha level of individual terms.

We selected the model that maximised explanatory power, minimised Akaike’s Information Criterion, minimised RMSE, and maximised convergence and stability measures.

**References**
