# Supplementary material for: Silent voices: Uncovering women’s absence in veterinary surgery publications
Source: PLoS One. 2025 Aug 14;20(8):e0330392. doi: 10.1371/journal.pone.0330392 (PMC12352642; doi:10.1371/journal.pone.0330392)
Supplement: Attachment — Submitted filename: PONE-D-25-22058_R.pdf [file pone.0330392.s002.pdf]

The study presents important and underexplored issues in veterinary academia with commendable rigor and transparency. With clearer acknowledgment of methodological and conceptual limitations, deeper engagement with systemic publication structures, and a slightly more critical tone toward underlying assumptions, this manuscript could make a high-impact contribution to gender equity scholarship in veterinary medicine.

Abstract:

- Lacks mention of limitations in methods (e.g., use of Gender API or binary gender framework).
- The sample size and timeframe are mentioned, but statistical model type (frequentist and Bayesian) could be more concisely stated.
- "Clinical Significance" feels slightly detached from the broader implications (which are more sociological than clinical).
- Consider rephrasing "clinical significance" to "professional significance" or "disciplinary significance" to reflect the academic nature of the issue.

Introduction:

- While comparisons to human medicine and STEMM are helpful, the veterinary-specific discussion could be deepened earlier.
- Some redundancy exists in restating general gender bias across multiple fields without narrowing focus on veterinary surgery until later.
- Consider streamlining general gender bias content to avoid duplication with the Discussion.

Material and methods:

- **Gender assignment** via name inference is problematic in a study on gender disparity. Though limitations are noted, this needs more prominence.
- No justification is given for excluding middle authors or focusing only on first, second, and last authors.
- No mention of any validation steps taken for manual gender identification (e.g., inter-rater agreement).
- Explicitly state the number/percentage of authors for whom gender could not be assigned.
- Clarify the rationale for choosing author order categories (why not include all authorship positions?).
- Recommend disclosing whether gender assignments were cross-checked by multiple reviewers for bias control.
- Acknowledge the potential for regional and language bias from use of name-based APIs (e.g., non-Western names).

Results:

- **R^2 = 0.05** indicates weak model explanatory power, which should be emphasized more clearly in the narrative. Emphasize that the low R^2 suggests important omitted variables.
- The categorization of surgical specialty (e.g., “Other”) could be more detailed or justified.
- Figure 2 is underexplained — the distinction between model-based vs observed trends may confuse readers without statistical background.

- Add a summary paragraph at the end of the Results synthesizing key trends and model insights in lay terms.

Discussion:
- Overreliance on external factors (e.g., culture, reputation) without tying them clearly to study findings.
- Discussion of author order and contribution is too accepting — it does not challenge the assumptions behind those roles.
- Discuss how journals or institutions can address disparities (e.g., mentorship, double-blind review, ORCID role tracking).
- Add a note about the role of editorial gatekeeping and how that may impact authorship trends.

Limitations:
- The language is cautious, but could be stronger in acknowledging how the limitations may bias findings.
- The limitation about excluding non-binary individuals is brief and insufficient given the topic.

Conclusion:
- The final paragraph implies causation (underrepresentation is *because* fewer women apply) where the data only shows association.
- Rephrase speculative causal statements (e.g., "likely because of") into hypothesis-generating observations.
- Consider emphasizing systemic and cultural reforms, not just training flexibility.
- Suggest follow-up studies (qualitative interviews, institutional policy audits, mentorship analysis).
